# Bioactivity of a Family of Chiral Nonracemic Aminobenzylnaphthols towards *Candida albicans*

**DOI:** 10.3390/molecules19045219

**Published:** 2014-04-23

**Authors:** Maria Annunziata M. Capozzi, Cosimo Cardellicchio, Angela Magaletti, Antonio Bevilacqua, Marianne Perricone, Maria Rosaria Corbo

**Affiliations:** 1 Department of Chemistry, University of Bari, Via Orabona 4, 70125 Bari, Italy; E-Mails: maria.capozzi@uniba.it (M.A.M.C.); angelamagaletti@gmail.com (A.M.); 2 CNR-ICCOM, at the Department of Chemistry, University of Bari, Via Orabona 4, 70125 Bari, Italy; E-Mail: cardellicchio@ba.iccom.cnr.it; 3 Department of the Science of Agriculture, Food and Environment, University of Foggia, Via Napoli 25, 71122 Foggia, Italy; E-Mails: marianne.perricone@unifg.it (M.P.); mariarosaria.corbo@unifg.it (M.R.C.)

**Keywords:** *Candida albicans*, Betti reaction, prolinol, anti-yeast activity, microdilution approach, interactive effects

## Abstract

Chiral nonracemic aminobenzylnaphthols were obtained by a Betti multi-component reaction between 2-naphthol, aryl aldehydes and enantiopure arylethylamine. Moreover, some new aminobenzylnaphthols were synthesized by a similar reaction between 2-naphthol, aryl aldehydes and prolinol. These aminobenzylnaphthols, synthesized from different components and thus having different structural features, were tested as anti-yeast agents inhibiting *Candida albicans*. The effect towards the test strain was studied with a microdilution approach and three different concentrations (150, 300 and 450 µg/mL) were tested. The best results were found for the aminobenzylnaphthols obtained from 1-naphthylethylamine and from natural prolinol. The use of the two-way ANOVA highlighted the better performances of the prolinol derivative among the differently structured aminobenzylnaphthols that were screened. The activity towards *C. albicans* of this prolinol derivative resulted to be interesting and could represent a promising alternative to overcome the problem of the strains resistant to the traditional antifungals.

## 1. Introduction

*Candida* spp., namely *C. albicans*, are one the most important causes of hospital acquired systemic infections, with crude mortality rate of up 50% [1,2]. These yeasts can cause two types of pathologies: superficial (oral and vaginal candidiasis) and life-threatening systemic infections [3]. *C. albicans*, and to a lesser extent the other species of the genus, can be easily found in the oral cavity of up to 75% of the population [3]. In healthy individuals this colonization remains benign; however, *C. albicans* could pose a strong threat in immunocompromised patients, who can suffer recalcitrant infections on the skin and in the oral cavity [4]. The rise in the number of immunocompromised patients has caused a dramatic increase of the incidence of fungal infections due to *C. albicans* and other related human opportunistic pathogens [5,6] and this issue is a great challenge for human health due also to the fact that some strains of these yeasts are resistant to some traditional antifungals, like the azoles [7].

Some alternatives to the azoles are extracts from plants (like the oils from *Iamium album*, *Cuminum cyminum*, *Salvadora persica*) [8,9], the combination of terpenes and fluconazole [10], bioremediation [11], as well as other agents [12,13].

**Scheme 1:**
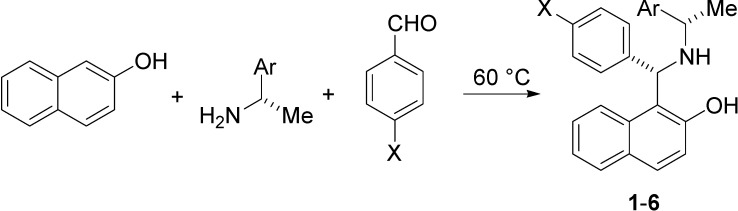
Synthesis of aminobenzylnaphthols **1**–**6**.

**Scheme 2:**
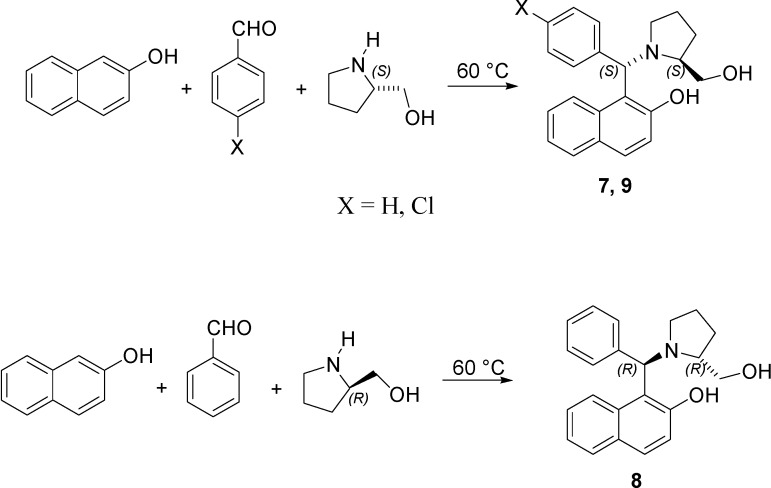
Synthesis of aminobenzylnaphthols **7**–**9**.

Previously, some papers [14,15,16,17,18,19] reported the Betti reaction as a route for the asymmetric synthesis of chiral nonracemic amino benzylnaphthols. This procedure is a straightforward condensation of 2-naphthol, aryl aldehydes and suitable chiral nonracemic amines (Scheme 1 and Scheme 2) to yield more complex molecular structures. When an enantiopure (*S*)-amine was used, a (*S*,*S*)-aminobenzylnaphthol was easily isolated [14,20].

The experimental procedure that was applied [14] to the synthesis of aminobenzylnaphthols **1**–**6** (Scheme 1) was extended in the present paper to the reaction of 2-naphthol, aryl aldehyde and (*S*)- or (*R*)-prolinol (Scheme 2), to yield the corresponding aminobenzylnaphthols **7**–**9**.

**Table:** 

Compound	X	Ar
(*S*, *S*)-1	H	Ph
(*S*, *S*)-2	F	Ph
(*S*, *S*)-3	Cl	Ph
(*S*, *S*)-4	H	1-Np
(*S*, *S*)-5	F	1-Np
(*S*, *S*)-6	Br	1-Np

**Table:** 

Compound	X	Amine
(*S*, *S*)-7	H	(*S*)-Prolinol
(*R*, *R*)-8	H	(*R*)-Prolinol
(*S*, *S*)-9	Cl	(*S*)-Prolinol

At this stage, taking into account that *C. albicans* is a great threat for human health and the production of novel compounds with a significant bioactivity towards this yeast is an important issue for both organic chemistry and medicine, we decided to test the bioactivity of aminobenzylnaphthols **1**–**9** against a collection strain of *C. albicans* to correlate the results of the screening to the structural features of the investigated molecules.

## 2. Results and Discussion

The experiments were performed on aminobenzylnaphthols divided into three main groups, depending on the enantiopure amine that was employed in the Betti reaction (Scheme 1 and Scheme 2). In particular, compounds **1**–**3** were synthesized having (*S*)-phenylethylamine as a precursor; compounds **4**–**6** were obtained starting from (*S*)-1-(1-naphthyl)ethylamine; finally, compounds **7**–**9** were the new aminobenzylnaphthols related to the prolinol structure.

When testing the bioactivity of a chemical, the choice of the type of approach is a critical step; namely, the evaluation of the antimicrobial activity relies upon two main approaches: the agar diffusion and the microdilution test. In this paper, we utilized the microdilution method, due to its suitability to assess the Minimal Inhibitory Concentration and to establish an eventual time-kill response. Moreover, due to a possible interference of DMSO in the evaluation of the anti-yeast activity [21], two types of controls were prepared and respectively, analyzed (without and with the solvent, samples C1 and C2, respectively). In all the experiments the differences in cell counts with and without this solvent were generally not significant (*P* > 0.05, one-way ANOVA, data not shown), with some exceptions to this generalized statement.

Table 1 shows the anti-yeast effect of the compounds **1**–**3** belonging to the first group. An anti-yeast effect was observed only after 6 h, with a decrease in yeast count of 0.5 log cfu/mL (cfu, colony forming units); however, this activity was not related to the amount of the compounds in the broth, as it was found for the lowest concentrations (150 and 300 μg/mL) of the molecules **2** and **3** but not for the highest one (450 μg/mL). This result could be due to the low solubility of the investigated compounds **1**–**3** in aqueous systems. Thus, we could assume that the lower bioactivity at the highest concentration was probably due a partial dissolution of the compounds in the broth, resulting in a lower active concentration.

**Table 1:** Anti-yeact activity ([count in the control C2] − [count in the samples containing the chemicals]) (log cfu/mL; cfu, colony forming units) of the compounds **1**–**3** towards *C. albicans* in YPG broth. The letters indicate the significant differences (one-way ANOVA and Tukey’s test, *P* < 0.05). The symbol “/” indicate a not significant effect.

Samples/ concentrations	Time (h)	
	6	24
(*S*,*S*)-**1**		
150 μg/mL	^/^	^/^
300 μg/mL	^/^	^/^
450 μg/mL	^/^	^/^
(*S*,*S*)-**2**		
150 μg/mL	0.50 ^a^	^/^
300 μg/mL	0.55^ a^	^/^
450 μg/mL	^/^	^/^
(*S*,*S*)-**3**		
150 μg/mL	0.53 ^a^	^/^
300 μg/mL	0.55 ^a^	^/^
450 μg/mL	^/^	^/^

**Figure 1:**
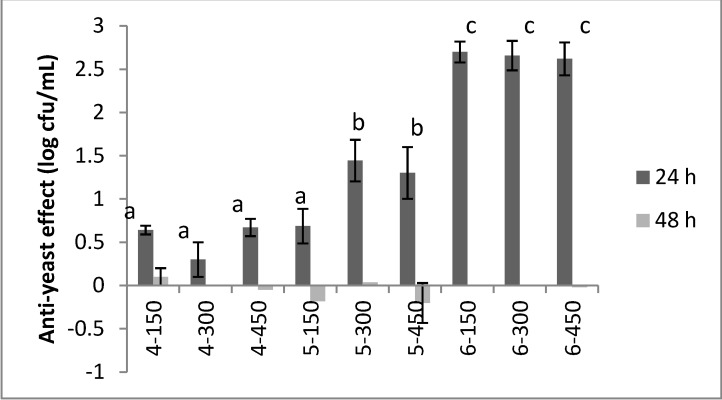
Anti-yeast effect of the compounds **4**–**6** after 24 and 48 h. The results were reported as difference between the yeast count in the control C2 and the level of the target in the samples containing the antimicrobial compounds. Mean values ± standard deviation. The letters indicate significant differences (one-way ANOVA and Tukey’s test, *P* < 0.05). The number after the compounds indicate its concentration in the broth (150, 300 or 450 µg/mL).

**Figure 2:**
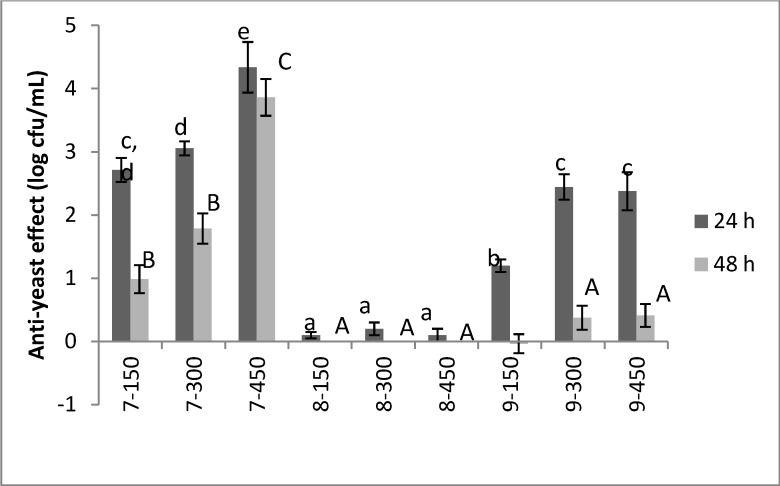
Anti-yeast effect of the compounds **7**–**9** after 24 and 48 h. The results were reported as difference between the yeast count in the control C2 and the level of the target in the samples containing the antimicrobial compounds. Mean values ± standard deviation. The letters indicate significant differences (one-way ANOVA and Tukey’s test, *P* < 0.05) (Small letters: significant differences after 24 h; Capital letters; significant differences after 48 h). The number after the compounds indicate its concentration in the broth (150, 300 or 450 µg/mL).

A significant bioactivity was found for the compounds of the 2nd (**4**–**6**) and 3rd series (**7**–**9**), as reported in Figure 1 and Figure 2. Concerning the group of molecules **4**–**6**, their bioactivity was strongly affected by the structure, the concentration and the time. In particular, compound **4** reduced the count of *C. albicans* by *ca*. 0.5 log cfu/mL and the effect of the concentration was not significant. On the other hand, the concentration played a significant role for the compound **5**, as it reduced the yeast by 0.68 ± 0.2 log cfu/mL at 150 µg/mL and 1.33–1.44 log cfu/mL at 300–450 µg/mL. Finally, the strongest bioactivity was observed for the molecule **6**, as it caused a decrease of the yeast by 2.7 log cfu/mL. After 48 h the compounds of this group did not exert any type of anti-yeast effect.

As regards to the bioactivity of the molecules of the third group **7**–**9**, after 24 h the effect of the compound **7** was strong and relied upon the concentration, as it reduced *C. albicans* by 2.7–3.0 log cfu/mL at 150/300 µg/mL and 4.34 ± 0.40 log cfu/mL at 450 µg/mL. Its stereoisomer **8**, synthesized starting from the unnatural (*R*)-prolinol, did not exert any effect towards *C. albicans*. An interesting and significant bioactivity was found for the compound **9**, which decreased the yeast by 1.20 ± 0.10 log cfu/mL at 150 µg/mL and *ca*. 2.4 log cfu/mL at 300/450 µg/mL. After 48 h, a significant effect was recovered only for the compound **7**, which reduced the level of *C. albicans* by 1–3.68 log cfu/mL (Figure 2).

As a final step of this research, the effects of the molecules tested in the first run and able to reduce the level of *C. albicans* (*i.e.*, **5**, **6**, **7**, and **9**) were used as input values for a two-way ANOVA to highlight the most promising compound. Two way ANOVA could be described as the most simple factorial analysis; it is based on the analysis of variance, with an important difference: the results can be classified by two factors (in this paper the kind of the compound and the concentration) and the analysis can highlight the differences due to each factor and the variability linked to their interaction.

**Figure 3:**
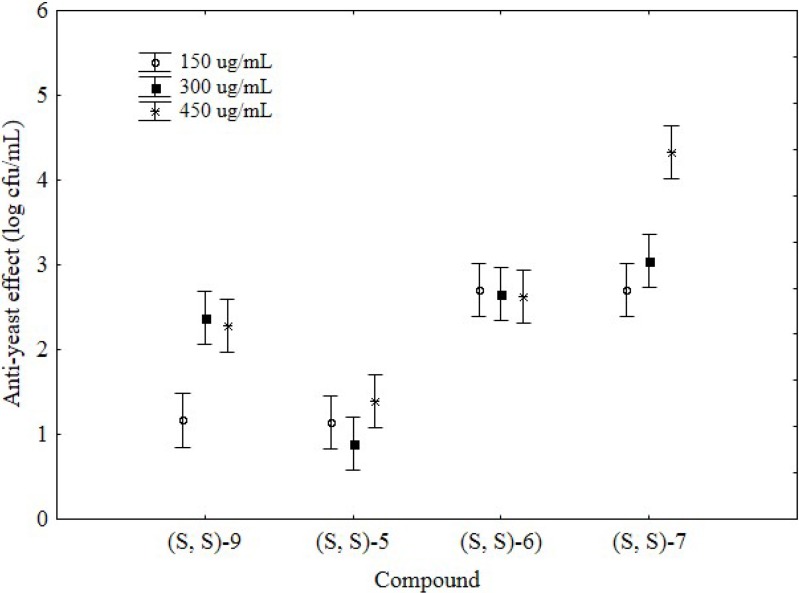
Decomposition of two-way ANOVA for the effects of the kind of the chemical and the concentration (comparison of the most promising compounds from the second and the third groups); the bars denote 95%-confidence intervals.

The main output of this analysis are the table of the statistical effects and the hypothesis decomposition; the table of the effects reports a qualitative output, *i.e.*, if a factor is significant or not, whilst the graph of hypothesis decomposition shows the quantitative effect. Table 2 shows the statistical effect of the analysis, whereas Figure 3 reports the hypothesis decomposition.

**Table 2:** Two-way ANOVA for the effects of the kind of chemicals and their concentration on the anti-yeast effect after 24 h: comparison of the most promising compounds from the second group (**4**–**6**) and the third groups (**7**–**9**). SS, sum of squares; df, degrees of freedom; MS, mean square; F, Fisher test value.

Effects	SS	df	MS	F	*p*-value
Compound	24.6148	3	8.20494	119.3	1.49E-14
Amount	3.17882	2	1.58941	23.1	2.55E-06
Interaction	4.30922	6	0.7182	10.44	1.03E-05
Within	1.65102	24	0.06879		
Total	33.7539	35			

The statistical analysis pointed out a significant effect of the two factors (compound and concentration); moreover, the ANOVA highlighted a significant interaction compound/concentration. The hypothesis decomposition graph shows this type of interaction for compounds **9** and **7**; namely the effects of compound **9** were strengthened at 300 µg/mL, whilst molecule **7** underwent to a significant increase of its activity at 450 µg/mL. Concerning the most promising compound, the highest bioactivity was found for compound **7** at 450 µg/mL; moreover, this molecule also retained its effect after 48 h.

## 3. Experimental Section

### 3.1. Strain

*Candida albicans* DSM 70014 (DSMZ, Deutsche Sammlung von Mikroorganismen und Zellkulturen, Braunschweig, Germany) was used as the test strain; the yeast was stored at 4 °C on YPG slants (yeast extract, 10 g/L; bacteriological peptone, 20 g/L; glucose, 20 g/L; agar, 15 g/L) and grown before each assay in YPG broth, incubated at 25 °C for 48–72 h. Thereafter, yeast cultures were centrifuged at 1,000 g for 10 min, the supernatant was discarded and the pellet suspended in sterile saline solution (0.9% NaCl); yeast suspension was diluted to *ca.* 5 log cfu/mL.

### 3.2. Tested Molecules

Starting materials for the Betti reaction were purchased from Sigma–Aldrich (Milan, Italy) and were used as received. NMR spectra were recorded on a Bruker AM500 spectrometer. HPLC analyses were performed with an Agilent 1100 chromatograph, equipped with a DAD detector. Scheme 1 and Scheme 2 show the different aminobenzylnaphthols used in this research.

#### 3.2.1. Synthesis of Aminobenzylnaphthols

(*S*,*S*)-Aminobenzylnaphthols **1**–**6** were synthesized [14] according to the previously reported procedure by reacting 2-naphthol, aryl aldehydes and (*S*)-1-arylethylamine (Scheme 1). Physical and spectral properties of the compounds **1**–**3** and **5**–**6** were already reported in the same paper [14]. Physical and spectral properties of aminobenzylnaphthol **4** were reported in a different work [22].

Aminobenzylnaphthols **7**–**8** (Scheme 2) were synthesized by reacting 2-naphthol, aryl aldehydes and *(S)*- or *(R)*-prolinol for two days at 60 °C without any solvent. After this time, the crude reaction mixture was cooled to room temperature and purified by column chromatography (eluent *n*-hexane/ethyl acetate 7:3), followed by crystallization. When (*S*)-prolinol was used, two compounds, whose NMR spectra are compatible with the (*S*,*S*) and (*R*,*S*)-aminobenzylnaphthol **7**, were recovered. Among them, the most abundant isomer was fully characterized and later used for the biological tests.

#### 3.2.2. 1-[(2-Hydroxymethyl-pyrrolidin-1-yl)-benzyl]-naphth-2-ols **7** and **8**—Predominant Stereoisomer

**Figure 4:**
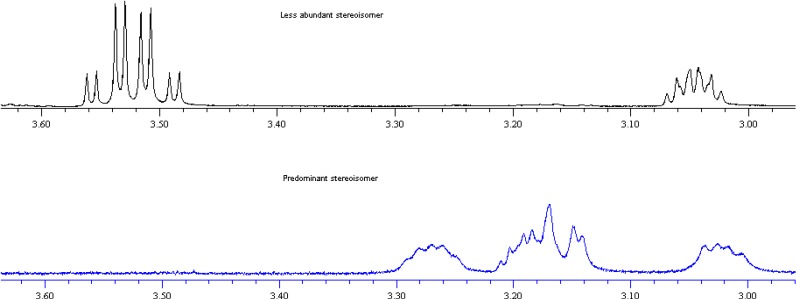
NMR spectra of aminobenzylnaphthols **7/8**.

Isolated yield: 37%. Mp 138–140 °C (ethanol). [α]_D_^25^ (c = 0.7, chloroform) = +199 for **7** and −192 (c = 0.5, chloroform) for **8**. ^1^H-NMR (CDCl_3_, 500 MHz) δ 14.03–13.86 (broad, 1 H), 7.82–7.78 (m, 1 H), 7.72–7.67 (m, 2 H), 7.64–7.60 (m, 2 H), 7.37–7.33 (m, 1 H), 7.30–7.26 (m, 2 H), 7.25–7.20 (m, 2 H), 7.17 (d, *J* = 8.9 Hz, 1 H), 5.44 (s, 1 H), 3.30–3.22 (m, 1 H), 3.21–3.11 (m, 2 H), 3.06–2.98 (m, 1 H), 2.69–2.62 (m, 1 H), 2.10–2.01 (m, 1 H), 1.93–1.83 (m, 3 H), 1.72–1.41 (broad m, 1 H). ^13^C-NMR (CDCl_3_, 125 MHz) δ 155.6, 140.2, 131.8, 129.6, 129.4, 129.0, 128.9, 128.8, 128.6, 128.3, 126.4, 122.4, 120.9, 120.0, 70.2 (broad), 64.8, 61.8, 55.9 (broad), 28.5, 23.8. In the absence of a crystal suitable for the X-ray analysis, the configuration of the newly formed stereogenic centre of the aminobenzylnaphthols **7** and **8** was attributed by the following combined NMR and computational analysis. The ^1^H-NMR spectra of the couple of diastereomeric aminobenzylnaphthols **7** (predominant and less abundant stereoisomer) isolated from the reaction mixture were compared. The overlap of a section of the two NMR spectra is illustrated in Figure 4.

As can be seen, the signals of the methylene hydrogen atoms of the hydroxymethyl moiety in the case of less abundant stereoisomer of **7** are centered at 3.52 ppm (Figure 4, top). On the other hand, in the case of the predominant stereoisomer of **7**, the same signals move upfield to coalesce in the 3.21–3.11 ppm multiplet (Figure 4, bottom).

**Figure 5:**
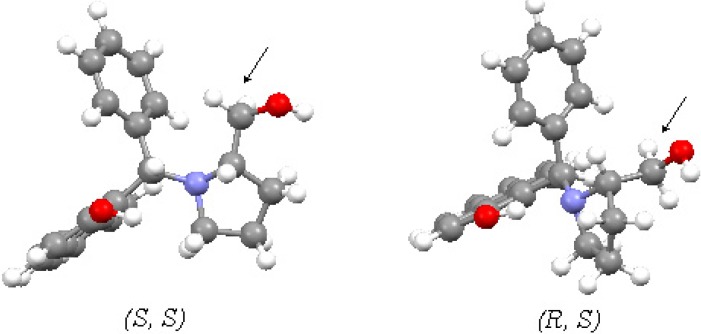
Spatial representation of aminobenzylnaphthols **7/8**.

The structures of the two different stereoisomers (*R*,*S*)-**7** and (*S*,*S*)-**7** were optimized with an HF-calculation (Spartan program), with the further constrain of a hydrogen bond between the naphthol hydrogen atom and the nitrogen atom, fixed at the average value of 1.85 Å, as occurred in every other aminobenzylnaphthols [14,17]. Within this constrain, the calculation shows that the exocyclic methylene group points towards the phenyl moiety in the case of the (*S*,*S*)-aminobenzylnaphthol **7**, whereas the same group points towards free space in the case of the (*R*,*S*)-counterpart (Figure 5).

In a constrained structure, if some hydrogen atoms points towards an aryl group, an upfield shift of their ^1^H-NMR signals is observed. Since this upfield shift was predicted for the (*S*,*S*)-configuration and observed for the predominant stereoisomer, then the (*S*,*S*)-configuration can be attributed to the most abundant stereoisomer. In summary, when natural (*S*)-prolinol was used in the Betti reaction, (*S*,*S*)-aminobenzylnaphthol **7** was mainly obtained, in complete agreement with the observed stereochemical course of the Betti reactions reported to date. Conversely, when unnatural (*R*)-prolinol was used in the Betti reaction, (*R*,*R*)-aminobenzylnaphthol **8** was synthesized.

A chiral HPLC analysis (column Chiralcel OD-H, eluent *n*-hexane/*i*-propanol 9:1) was performed also on the (*S*,*S*)-aminobenzylnaphthols **7** and (*R*,*R*)-**8** to check that no racemization at the stereogenic centre deriving from the (*S*)-prolinol occurred during the reaction. The separation conditions had been preliminarily optimized with a simulated racemic mixture. The chiral HPLC analysis confirmed that no racemization was observed.

#### 3.2.3. 1-[(4-Chlorophenyl)-(2-hydroxymethylpyrrolidin-1-yl)-methyl]-naphth-2-ol (**9**)

This compound was obtained using the experimental conditions outlined for compounds **7** and **8**. Isolated yield: 36%. Mp 75–77 °C (ethanol). [α]_D_^25^ (c = 0.25, chloroform) = +83. ^1^H-NMR (CDCl_3_, 500 MHz) δ 13.86–13.74 (broad, 1 H), 7.76–7.64 (m, 3 H), 7.57–7.52 (m, 2 H), 7.37–7.31 (m, 1 H), 7.26–7.19 (m, 3 H), 7.13 (d, *J* = 9.2 Hz, 1 H), 5.41 (s, 1 H), 3.27–3.01 (m, 4 H), 2.66–2.57 (m, 1 H), 2.08–1.98 (m, 1 H), 1.91–1.80 (m, 3 H), 1.54–1.34 (broad, 1 H). ^13^C-NMR (CDCl_3_, 125 MHz) δ 155.5, 138.8, 134.1, 131.6, 130.7, 129.9, 129.0, 128.6, 126.6, 122.5, 120.6, 120.0, 116.4, 69.4 (broad), 64.7, 61.6, 55.7 (broad), 28.3, 23.8. The (*S*,*S*)-configuration of aminobenzylnaphthol **9**, prepared starting from (*S*)-prolinol, was attributed by analogy to the previously cited combined NMR and computational analysis. Also in this case, the chiral HPLC analysis (Chiralcel OD-H column; eluent: *n*-hexane/*i*-propanol 9:1) confirmed that no racemization occurred during the Betti reaction.

### 3.3. Evaluation of the Anti-Yeast Activity

The bioactivity of the aminobenzylnaphthols was assessed through a microdilution approach, modified as follows. Aliquots of YPG broth (9.3 mL) were inoculated with *C. albicans* to 3 log cfu/mL and added with variable amounts of DMSO and/or of the stock solutions; the preparation of the samples is shown in Table 3. Two different kinds of control were prepared: C1, without the chemicals and DMSO, and C2, containing DMSO but not the molecules. A stock solution of each chemical was prepared by solving 7.5 g of the compound *per* liter of DMSO (dimethyl sulfoxide).

**Table 3:** Preparation of the sample to assess the bioactivity of the molecules through the microdilution approach.

Samples	Stock solution (mL)	DMSO (mL)	Saline solution (mL)	Inoculum (mL)
150 μg/mL	0.2	0.4	-	0.1
300 μg/mL	0.4	0.2	-	0.1
450 μg/mL	0.6	-	-	0.1
Control 1 (C1)	-	-	0.6	0.1
Control 2 (C2)	-	0.6	-	0.1

The samples were stored at 25 °C and the viable count of *C. albicans* was assessed periodically (after 6, 24 and 48 h) through the spread plating on YPG plates, incubated at 25 °C for 48–72 h.

The microbiological analyses were performed in duplicate over two different batches; the results were analyzed through the one-way and two-way Analysis of Variance and Tukey’s test as the *post-hoc* comparison test (*P* < 0.05) using the software Statistica for Windows ver. 10.0 (Statsoft, Tulsa, OK, USA).

## 4. Conclusions

The increase of the incidence of the strains of *C. albicans* resistant to some traditional antifungals is a great threat for human health; however, the multicomponent Betti reaction could be a promising and simple way to design novel compounds to inhibit this yeast. The results of this paper suggest that the bioactivity of aminobenzylnaphthols synthesized by this reaction could be affected by different factors, connected to the different components of the Betti procedure:

1. The structure of the compounds, since biological activity was detected for aminobenzyl-naphthols synthesized starting from 1-(1-naphthylethylamine) or from prolinol (groups 2 and 3), but not observed for molecules derived from 2-phenyethylamine.
2. The presence on the tested molecules of the halogen atom in the *para*-position of the phenyl group deriving from the aryl aldehyde employed in the Betti reaction, since chlorine or bromine atom can affect the bioactivity.
3. The stereochemistry of the amine used in the Betti reaction, since the aminobenzylnaphthols deriving from the natural (*S*)-prolinol showed a strong effect towards *C. albicans*, whilst the compound prepared by the unnatural (*R*)- prolinol does not.

As a final step of this research, a promising compound was selected, *i.e.*, (*S*,*S*)-1-[(2-hydroxymethyl-pyrrolidin-1-yl)-benzyl]-naphth-2-ol or (*S*,*S*)-**7**, that is able to reduce by 4.34 log cfu/mL the count of *C. albicans* at 450 µg/mL after 24 h and to retain a significant effect also after 48 h. A future trend of this research could be the application of this compound as a tool to disrupt or control biofilms formed by *Candida* spp.
